# Severity of diabetic retinopathy severity with renal function and body fat in type 2 diabetes mellitus: A hospital-based cross-sectional study in India

**DOI:** 10.6026/973206300213990

**Published:** 2025-10-31

**Authors:** Alwin Kurian Raju, Jojo Pullockara1, Thomas Cheriyan

**Affiliations:** 1 Department of Nephrology, Apollo Adlux Hospital, Karukkuty, Cochin, Kerala, India; 2 Department of Ophthalmologist, Retina and Uvea Specialist, Little Flower Hospital and Research Centre, Angamaly, Kerala, India

**Keywords:** Diabetic retinopathy, renal dysfunction, body fat percentage, type 2 diabetes mellitus, skinfold thickness, durnin-womersley method, microvascular complications, anthropometry, risk stratification, cross-sectional study

## Abstract

Diabetic retinopathy (DR) severity and renal impairment are important complications of type 2 diabetes mellitus. However, the relationship between them and body fat percentage (BFP) is not known. Hence, a cross-sectional study was conducted on 166 patients aged 30-70 years that measured DR severity, renal function and body fat percentage (BFP) using traditional clinical and anthropometric assessments. This data shows DR severity was related to measurable deterioration in renal parameters and raised body fat percentage (BFP). Thus, we show the understanding of the inter-relationships between microvascular and metabolic-related complications of diabetes. Increased screening earlier may help risk stratify and potentially facilitate comprehensive management of the patient in the clinical setting.

## Background:

Diabetes mellitus (DM) is a long-lasting metabolic condition that is noted for sustained hyperglycemia that significantly contributes to a range of microvascular and macrovascular complications later on. Diabetic retinopathy (DR) is perhaps the most common microvascular complication of DM and it remains a leading cause of preventable blindness for working-age adults around the world impacting quality of life and productivity [1]. DR develops because of prolonged exposure to hyperglycemia that leads to endothelial injury, pericyte loss, thickening of capillary basement membranes and ultimately occlusion of the small vessels leading to retinal ischemia and neovascularization [2]. With hyperglycemia, the presence of hypertension, dyslipidemia and other metabolic derangements may contribute to the presence and progression of both DR and systemic damage to microvessels [2]. Population-based studies such as the Early Treatment Diabetic Retinopathy Study (ETDRS) and the Wisconsin Epidemiologic Study of Diabetic Retinopathy (WESDR) demonstrated the clinical significance of measuring gradings of DR severity. The features most predictive of progression of DR to more severe form of DR and vision prognosis were the presence of microaneurysms, intraretinal microvascular abnormalities (IRMA), hemorrhages and neovascularization [3]. The presence of eye-related consequences for dysregulated microcirculation may also track damage to the microvasculature systematically with diabetic nephropathy being a similar consequence of the underlying pathophysiology of injury to the endothelium, pericyte loss, capillary rarefaction and chronic metabolic burden [4]. Renal function indicators such as serum creatinine, estimated glomerular filtration rate (eGFR) and electrolyte profiles are associated with DR severity, demonstrating the interconnectedness of microvascular tissue damage in diabetes [5]. In addition to renal and the ocular complications, change in body composition, especially increased visceral adiposity, also plays an important contributor to insulin resistance, chronic inflammation and metabolic dysregulation. Visceral fat accumulation stimulates proinflammatory cytokine type release, along with oxidative stress and dyslipidemia, which may accelerate microvascular injury and increase DR severity [6]. Anthropometric methods such as the Durnin-Womersley four-site skinfold thickness, is a valid non-invasive method to measure percentage body fat, which allows clinicians to stratify metabolic risk and facilitate the management of interventions in diabetic populations [7]. Although there is considerable evidence linking DR, renal disease and adiposity individually, there is limited research that examines direct evidence of them together (in research populations, particularly in India). An understanding of the interrelationships is important for the identification of high-risk patients to initiate management and there is an opportunity to leverage such relationships that may facilitate a combined assessment approach for ocular, renal and metabolic health. This applies to a population with significantly high prevalence of type 2 diabetes - accompanied by increased prevalence of obesity/overweight and metabolic syndrome - with DR and the associated complications of high burden becoming apparent [8]. Therefore, it is of interest to describe the correlation between diabetic retinopathy severity, renal function parameters and body fat percentage in patients with type 2 diabetes mellitus, with the aim of informing holistic risk assessment and management strategies in clinical practice.

## Materials and Methods:

This hospital-based cross-sectional observational study was conducted over six months at a tertiary care center. Patients attending the outpatient and inpatient departments, aged between 30 and 70 years and diagnosed with type 2 diabetes mellitus were considered for inclusion. Patients provided written informed consent before enrollment. Those with type 1 diabetes, chronic kidney disease (stage 4 or higher), pregnancy, lactation, ocular trauma, or recent intraocular surgery were excluded. A total of 166 participants were enrolled using convenience sampling. Comprehensive demographic and clinical details, including duration of diabetes, hypertension history, medications, smoking and alcohol intake, were recorded using a structured case record form (CRF). All subjects underwent detailed ophthalmic examinations performed by a single trained ophthalmologist. Diabetic retinopathy severity was classified using the International Clinical Diabetic Retinopathy Severity Scale, encompassing categories from no apparent retinopathy through proliferative diabetic retinopathy (PDR). Indirect ophthalmoscopy was carried out post pupillary dilation, enabling standardized and reproducible retinal assessment. Renal parameters were assessed by measuring serum creatinine (mg/dL), blood urea (mg/dL) and serum electrolytes, including sodium (mEq/L) and potassium (mEq/L). The estimated glomerular filtration rate (eGFR, mL/min/1.73 m^2^) was calculated using the Modification of Diet in Renal Disease (MDRD) equation. Laboratory values were obtained from routine clinical reports and fresh samples were collected when recent laboratory data was unavailable.

Anthropometric evaluation involved measurements of weight (kg), height (cm) and calculation of body mass index (BMI, kg/m^2^). Body fat percentage was estimated using the Durnin-Womersley method, utilizing skinfold thickness measurements at four standardized anatomical sites: triceps, biceps, subscapular and suprailiac regions. Measurements were performed with calibrated Harpenden skinfold calipers by a trained technician following the recommended procedure. The sum of these four skinfold measurements was logged and body density was computed based on age- and sex-specific regression equations of the Durnin-Womersley protocol. Subsequently, body fat percentage was determined using the Siri equation. Statistical analysis was conducted using SPSS software (version 25.0). Descriptive statistics summarized baseline characteristics, while continuous variables were expressed as means ± standard deviation or medians and interquartile ranges as appropriate. Categorical variables were presented as frequencies and percentages. Pearson's or Spearman's correlation tests, as indicated by data distribution, examined relationships among DR severity, renal parameters and body fat percentage. Comparisons across DR severity groups utilized ANOVA or Kruskal-Wallis tests, as appropriate, with significance established at p<0.05. Ethical clearance was obtained from the Institutional Ethics Committee prior to study initiation. Confidentiality and anonymity of patient data were strictly maintained. The study was conducted in compliance with the Declaration of Helsinki; ensuring participants incurred no additional risk or financial burden.

## Results:

A total of 166 participants with type 2 diabetes mellitus were enrolled in this study. The mean age of participants was 54.2 ± 9.6 years, with a predominance of males (73.5%). The mean duration of diabetes was 8.4 ± 5.2 years and hypertension was present in 80.7% of patients. Anthropometric parameters indicated a mean BMI of 27.6 ± 4.5 kg/m^2^, with a mean body fat percentage of 32.1 ± 6.7% estimated through skinfold thickness measurements. The results indicate clear and statistically significant correlations between diabetic retinopathy severity, renal function impairment and increased body fat percentage, suggesting their concurrent role in the progression of diabetic microvascular complications.

Table 1 (see PDF) presents the baseline characteristics of the 166 enrolled participants, showing a predominance of middle-aged males with a mean BMI of 27.6 kg/m^2^ and average body fat percentage of 32.1%. Table 2 (see PDF) outlines the distribution of diabetic retinopathy severity, revealing that nearly 44.6% of participants had mild to moderate NPDR, while 23.2% had advanced stages including severe NPDR and PDR. Table 3 (see PDF) shows a progressive deterioration in renal function across increasing DR severity, with rising serum creatinine and urea levels and a concurrent decline in eGFR values (p<0.001). Table 4 (see PDF) highlights a parallel rise in body fat percentage with DR progression, from 29.1% in patients without retinopathy to 36.8% in those with PDR. Table 5 (see PDF) illustrates strong correlations between diabetic retinopathy severity and renal parameters (positive with creatinine and urea; negative with eGFR) and body fat percentage, all significant statistically (p<0.001). These observations suggest a close, interdependent association between the severity of diabetic retinopathy, renal impairment and elevated adiposity.

## Discussion:

This investigation examined the interdependency between diabetic retinopathy severity, renal impairment and percentage of body fat in individuals with type 2 diabetes mellitus. The results provide evidence of a persistent and statistically significant association among these three factors, highlighting the interconnected pathophysiologic processes underlying microvascular complications of diabetes [9]. Notably, in concordance with increasing DR severity, renal function declined parallelly while body fat percentage rose, indicating a common etiologic paradigm of chronic hyperglycemia, insulin resistance, vascular endothelial dysfunction and systemic inflammation. The positive correlation of DR severity with serum creatinine and urea and negative correlation with eGFR, supports earlier reports that renal impairment has a close correlation with advancing retinopathy [10]. Several studies have documented that reduced renal clearance is a marker of systemic microvascular injury, which also manifests in the retinal vasculature. Thus, renal parameters serve not only as markers of nephropathy but may also function as surrogates of retinal vascular compromise in long-standing diabetes [11]. The significant association between increasing body fat percentage and DR severity observed in this study underlines the metabolic component in DR progression. Excess adiposity is known to promote low-grade chronic inflammation, oxidative stress and adipokine imbalance, all of which contribute to microvascular endothelial damage [12]. The use of the Durnin-Womersley four-site skinfold thickness method provided a non-invasive, yet reliable estimation of body fat percentage, offering insights into the role of altered body composition in diabetic complications. Unlike BMI, which fails to distinguish lean mass from fat mass, body fat percentage offers a more precise reflection of metabolic risk [13]. These findings support a more integrated clinical approach in the management of diabetes; wherein routine ophthalmologic evaluations are complemented by monitoring renal function and body composition metrics [14]. Early identification of patients at risk for progressive retinopathy through these systemic indicators can facilitate timely intervention and delay vision-threatening outcomes. Additionally, the data advocate for lifestyle modifications and weight reduction strategies as part of a holistic strategy to mitigate DR progression [15]. This study's strengths include its well-characterized sample, standardized measurements for anthropometry and fundus grading and the use of validated body fat estimation methods. However, it is important to note certain limitations. The cross-sectional design precludes inference of causality and being a single-center study, the findings may not be generalizable to broader populations. Further longitudinal studies are warranted to elucidate the temporal dynamics of these associations and assess the impact of targeted interventions on DR progression. The present study highlights a significant and clinically meaningful association between diabetic retinopathy severity, renal dysfunction and body fat percentage. These findings underscore the need for integrated metabolic, renal and ocular assessments in routine diabetic care to improve prognostication and guide comprehensive management strategies.

## Conclusion:

There is a significant correlation between diabetic retinopathy severity, renal dysfunction and increased body fat percentage among patients with type 2 diabetes mellitus. Thus, we show the importance of integrated screening and management strategies targeting ocular, renal and metabolic health to prevent progression of diabetic complications.
